# Analysis of candidate genes for cleft lip ± cleft palate using murine single-cell expression data

**DOI:** 10.3389/fcell.2023.1091666

**Published:** 2023-04-24

**Authors:** Anna Siewert, Benedikt Reiz, Carina Krug, Julia Heggemann, Elisabeth Mangold, Henning Dickten, Kerstin U. Ludwig

**Affiliations:** ^1^ Institute of Human Genetics, University of Bonn, School of Medicine and University Hospital Bonn, Bonn, Germany; ^2^ FASTGenomics, Comma Soft AG, Bonn, Germany

**Keywords:** cleft lip with or without cleft palate, single-cell RNA sequencing (scRNA-seq), IRF6, craniofacial development, expression pattern, single-cell transcriptomics

## Abstract

**Introduction:** Cleft lip ± cleft palate (CL/P) is one of the most common birth defects. Although research has identified multiple genetic risk loci for different types of CL/P (i.e., syndromic or non-syndromic forms), determining the respective causal genes and understanding the relevant functional networks remain challenging. The recent introduction of single-cell RNA sequencing (scRNA-seq) has provided novel opportunities to study gene expression patterns at cellular resolution. The aims of our study were to: (i) aggregate available scRNA-seq data from embryonic mice and provide this as a resource for the craniofacial community; and (ii) demonstrate the value of these data in terms of the investigation of the gene expression patterns of CL/P candidate genes.

**Methods and Results:** First, two published scRNA-seq data sets from embryonic mice were re-processed, i.e., data representing the murine time period of craniofacial development: (i) facial data from embryonic day (E) E11.5; and (ii) whole embryo data from E9.5–E13.5 from the Mouse Organogenesis Cell Atlas (MOCA). Marker gene expression analyses demonstrated that at E11.5, the facial data were a high-resolution representation of the MOCA data. Using CL/P candidate gene lists, distinct groups of genes with specific expression patterns were identified. Among others we identified that a co-expression network including *Irf6, Grhl3* and *Tfap2a* in the periderm, while it was limited to *Irf6* and *Tfap2a* in palatal epithelia, cells of the ectodermal surface, and basal cells at the fusion zone. The analyses also demonstrated that additional CL/P candidate genes (e.g., *Tpm1, Arid3b, Ctnnd1, and Wnt3*) were exclusively expressed in *Irf6*+ facial epithelial cells (i.e., as opposed to *Irf6-* epithelial cells). The MOCA data set was finally used to investigate differences in expression profiles for candidate genes underlying different types of CL/P. These analyses showed that syndromic CL/P genes (syCL/P) were expressed in significantly more cell types than non-syndromic CL/P candidate genes (nsCL/P).

**Discussion:** The present study illustrates how scRNA-seq data can empower research on craniofacial development and disease.

## 1 Introduction

Molecular malfunctions during craniofacial development can lead to cleft lip ± cleft palate (CL/P). CL/P represents one of the most common of all birth defects, with a global prevalence of 1 in 700 live births (Mangold et al., 2011). Importantly, CL/P can present either as an isolated, non-syndromic phenotype (nsCL/P), or within the context of more complex malformation syndromes (syCL/P), in which additional features indicative of a developmental defect are observed. Although CL/P can be caused by deleterious mutations in single high penetrance genes (Cox et al., 2018; Bishop et al., 2020), a considerable fraction of its genetic architecture is attributable to common risk variants. Research suggests that environmental factors also contribute to CL/P, as part of its multifactorial etiology (Murray, 2002).

For nsCL/P, genome-wide association studies (GWAS) have identified multiple risk loci, and positional analyses of these loci have revealed promising candidate genes. For most of these genes, however, few data are available concerning the mechanism through which they affect the underlying functional processes of craniofacial development. One of the few exceptions to this is the *IRF6-GRHL3-TFAP2A* network, which has been shown to underlie diverse types of orofacial clefting, including CL/P and cleft palate only (Kousa et al., 2019). In addition to challenges associated with attributing causality to individual variants and genes, this lack of knowledge is also explained by the limited access to molecular data from relevant time points in humans, due to technical and ethical limitations.

Recently, single-cell RNA sequencing (scRNA-seq) has been performed on tissue from embryonic mice, generating systematic transcriptomic data sets at cellular resolution. This offers new avenues for the study of the tissue-specific expression of genes that underlie developmental phenotypes, including CL/P. Two resources of particular value in terms of CL/P are the Mouse Organogenesis Cell Atlas (MOCA; Cao et al., 2019), and facial data from embryonic mice that were reported in 2019 (Li et al., 2019). While MOCA encompasses the developmental time frame embryonic day (E) 9.5–13.5, the data from Li et al. provide a deeper insight into the transcriptome of facial structures at E11.5. Two important challenges associated with the use of scRNA-seq data are data accessibility and comparability, particularly when data are generated in different labs. The data of MOCA and Li et al. vary in terms of the level of processing, output types, and usability for the research community.

The aims of the present study were to (i) aggregate these scRNA-seq data from embryonic mice and provide this as a resource for the craniofacial community; and (ii) demonstrate the value of these data in terms of the investigation of the gene expression patterns of CL/P candidate genes. First, both of the selected data sets were re-analyzed using a joint computational pipeline. Second, different CL/P candidate gene sets were used to illustrate the potential of scRNA-seq data for deciphering the CL/P etiology. In particular, the expression patterns of CL/P candidate genes were assessed across the time period of craniofacial development, with the aim of placing them in their cell type-specific context. We specifically analyzed epithelial and mesenchymal cell types, which have been previously shown to be involved in CL/P (Ji et al., 2020). As an application example, we investigated co-expression of members of the *Irf6-Grhl3-Tfap2a* genetic pathway in epithelial cell sub-types and identified further genes with a potential *Irf6* interaction in these cells. Finally, potential expression differences in candidate genes for syCL/P and nsCL/P were investigated in order to test the hypothesis that during embryonic development, syCL/P candidate genes are expressed in more tissues than is the case for candidate genes for nsCL/P.

## 2 Materials and methods

### 2.1 Data sources

Two sets of single cell data on murine embryonic development were downloaded and analyzed using the same computational pipeline, which is described in detail in “Data analysis.” The first data set comprised single-cell gene expression data from 7,893 single cells from the lambdoidal junction, which were extracted from 4-5 mouse embryos at E11.5 (Li et al., 2019). The corresponding gene-count matrix was downloaded from the Gene Expression Omnibus (RRID:SCR_005012; accession number: GSM3867275). The data set was then re-analyzed using our in-house pipeline. The latter included stricter filtering parameters (see below), thus reducing the number of single cells used for analysis (7,249 cells in total) compared to the original study. The final facial data set included 25 cell clusters.

The second data set was MOCA, which was generated from whole embryonic mice (Cao et al., 2019). The MOCA data set comprises the expression data of 2,058,652 single cells, as obtained from 61 mouse embryos from developmental stages E9.5–E13.5. Post-filtering, the original data set contained data on 1,331,985 cells and 38 major cell types (Supplementary Table S1). The gene-count matrix containing these 1,331,985 pre-filtered, high-quality cells was downloaded from the MOCA Website, and stored and analyzed using FASTGenomics (Scholz et al., 2018; RRID:SCR_022898). In contrast to the original publication, the gene count matrix was split into five data sets in accordance with embryonic day in order to create a developmental time frame of gene expression: 112,269 cells (E9.5); 258,104 cells (E10.5); 449,614 cells (E11.5); 270,197 cells (E12.5); and 241,800 cells (E13.5).

### 2.2 Data analysis

#### 2.2.1 General processing

Each of the data sets was processed using the R package Seurat v4 (Hao et al., 2021; RRID:SCR_016341). To normalize the count matrices, Log normalization (normalization.method) was applied with a Seurat default scale factor of 10,000 (scale.factor). For the selection of highly variable genes, the “vst” selection method (selection.method) was chosen, using 2,500 as the number of features (nfeatures). Scaling was performed in block sizes of 500 (block.size). For linear dimension reduction, a principal component (PC) analysis was performed. To cluster the cells, a two-step approach was used. First, for each cell, the K-nearest neighbors were calculated using the *FindNeighbors* function of Seurat, based on the first 25 PC dimensions (dims). Second, the Louvain algorithm was applied as a modularity optimization technique with a resolution of 0.5 for MOCA data and 1.1 for facial data (resolution) using the *FindClusters* function. To identify differentially expressed genes (hereafter referred to as ‘marker genes’) for each cluster, the Wilcoxon Rank Sum test was used (test.use). Marker genes were obtained by comparing the expression levels of individual genes against all other clusters, and only positive markers were used. Additional parameters were a minimum fraction of 0.25 of cells expressing the tested gene in either of the populations (min.pct), and a threshold of a 0.25-fold change between the tested clusters (logfc.threshold). The uniform manifold approximation and projection algorithm (UMAP) was used as a non-linear dimension reduction method, whereby the first 25 PCs were applied as dimensions (dims).

#### 2.2.2 Study-specific filtering

For the facial data set, additional steps were performed pre-normalization. These included the filtering-out of potential doublets by excluding cells with >7,500 unique features (nFeature_RNA), and cells with >80,000 detected RNA molecules (nCount_RNA). To exclude cells that were previously lysed or apoptotic, cells with the presence of the following features were excluded from the data set: (i) a percentage of >5% of unique molecular identifiers reflecting mitochondrial genes (percent.mt); and/or (ii) < 2,300 unique features (nFeature_RNA). After filtering, our data set comprised 7,249 cells. To benchmark the present pipeline, cell type annotation was performed by comparing the marker genes of each cluster with the marker genes described in the original publication.

For the pre-filtered, high-quality cells of the MOCA data, no additional filtering was required. Final cell type annotation was performed using the published marker genes of Cao et al. and the R package scCATCH (Shao et al., 2020). For the latter, *species* was set to “Mouse”; *match_CellMatch* was set to “TRUE”; and the tissues selected to be matched to “CellMatch” were “Brain,” “Fetal brain” and “Embryo”. Further parameters were kept at default values.

### 2.3 Curation of CL/P candidate gene lists

**FIGURE 1:**
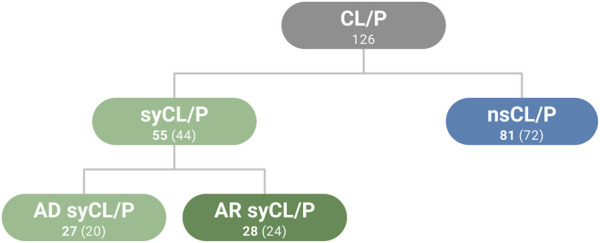
Summary of gene lists used in the present study. Genes that overlapped between categories are included in the numbers of genes indicated in bold (n = 10). Numbers in parentheses correspond to the number of unique genes in the respective category, without overlapping genes. CL/P (cleft lip with or without cleft palate), ns (non-sndromic), sy (syndromic), AD (autosomal dominant), AR (autosomal recessive).

A literature search was performed to generate lists of genes associated with non-syndromic and syndromic forms of CL/P. The nsCL/P gene list was generated based on a recent meta-analysis of nsCL/P GWAS (Welzenbach et al., 2021). Welzenbach et al. performed a gene-based analysis for genes located at established GWAS risk loci, which identified a set of 81 genes with an enrichment of common variants. These 81 genes were used in the present study. The syCL/P *gene list* was generated using information from a recently published study (Bishop et al., 2020), which had involved a systematic review of orofacial clefting syndromes and their associated genes. For the purposes of the present study, the list of syndromes generated by Bishop et al. was reduced using OMIM (RRID:SCR_006437) in order to: (i) include only those syndromes whose phenotype includes CL/P, with the exclusion of other orofacial clefting phenotypes; and (ii) generate subsets of genes with autosomal dominant (AD) or autosomal recessive (AR) contributions. An overview of the gene categories is provided in Figure 1. Genes that overlapped between the syndromic and non-syndromic categories were included in an ‘overlapping genes‘ list. Use of this list was restricted to the comparison of expression data between syCL/P and nsCL/P. The final numbers of unique genes for these analyses were 126 genes for CL/P overall, of which 72 genes were for nsCL/P, and 44 genes were for syCL/P (20 AD genes and 24 AR genes). Ten genes overlapped both categories.

To evaluate whether the findings for CL/P are generalizable to other birth defects, gene lists were also generated for congenital heart disease (CHD). A recent publication (Nees and Chung, 2020) listed 18 genes for non-syndromic CHD (nsCHD) and 56 genes for syndromic CHD (40 AD, 16 AR). Three genes overlapped both categories. However, this group was not analyzed in the present study due to the low number of genes. All gene lists are provided in Supplementary Table S2.

### 2.4 Creating Irf6+ and Irf6- epithelial cell sub-clusters

Based on its well-established role in both syCL/P and nsCL/P (Woude, 1954; Birnbaum et al., 2009), analyses were performed to investigate the role of *Irf6* in epithelial cells. To create *Irf6*+ and *Irf6*-epithelial sub-clusters, epithelial cell clusters in the facial data set (i.e., palatal epithelium, olfactory epithelium, ectodermal surface, ectodermal surface (*Robo2*+), periderm, and basal cells at the fusion zone) were divided into subsets according to *Irf6* expression. Previous research has shown that *Irf6*, *Grhl3,* and *Tfap2a* are part of a genetic network in which *Irf6* influences the gene expression of *Grhl3* and *Tfap2a* (Kousa et al., 2019). In order to examine if these genes are among the marker genes of the *Irf6*+ sub-clusters and to identify possible additional genes that are influenced by *Irf6*, we determined marker genes for these sub-clusters. For this purpose, the expression profiles of each sub-cluster were compared against all other cell clusters in the data set, using the parameters applied in the initial data analysis (see Data analysis; Supplementary Table S3).

### 2.5 Analysis of differences in gene expression between nsCL/P and syCL/P

The analysis of nsCL/P and syCL/P gene lists was performed in the whole embryo MOCA data sets. Two parameters were used in these comparisons: (i) the percentage of all cell types in which the respective genes were expressed; and (ii) the average expression level. For analysis (i), a cell type was considered to express a certain gene if the gene was expressed in at least 10% of cells. Percentages were determined for each gene in the respective list. The distributions were statistically compared using the Welch *t*-test. For analysis (ii), the average expression levels per cell type were extracted for each gene using the *AverageExpression* function from Seurat v4. The mean of these expression levels was then calculated per gene. A statistical comparison of the mean expression levels between both gene lists was performed using the Welch *t*-test.

## 3 Results

### 3.1 Facial-specific and whole embryo scRNA-seq data provide complementary insights into craniofacial development

**FIGURE 2:**
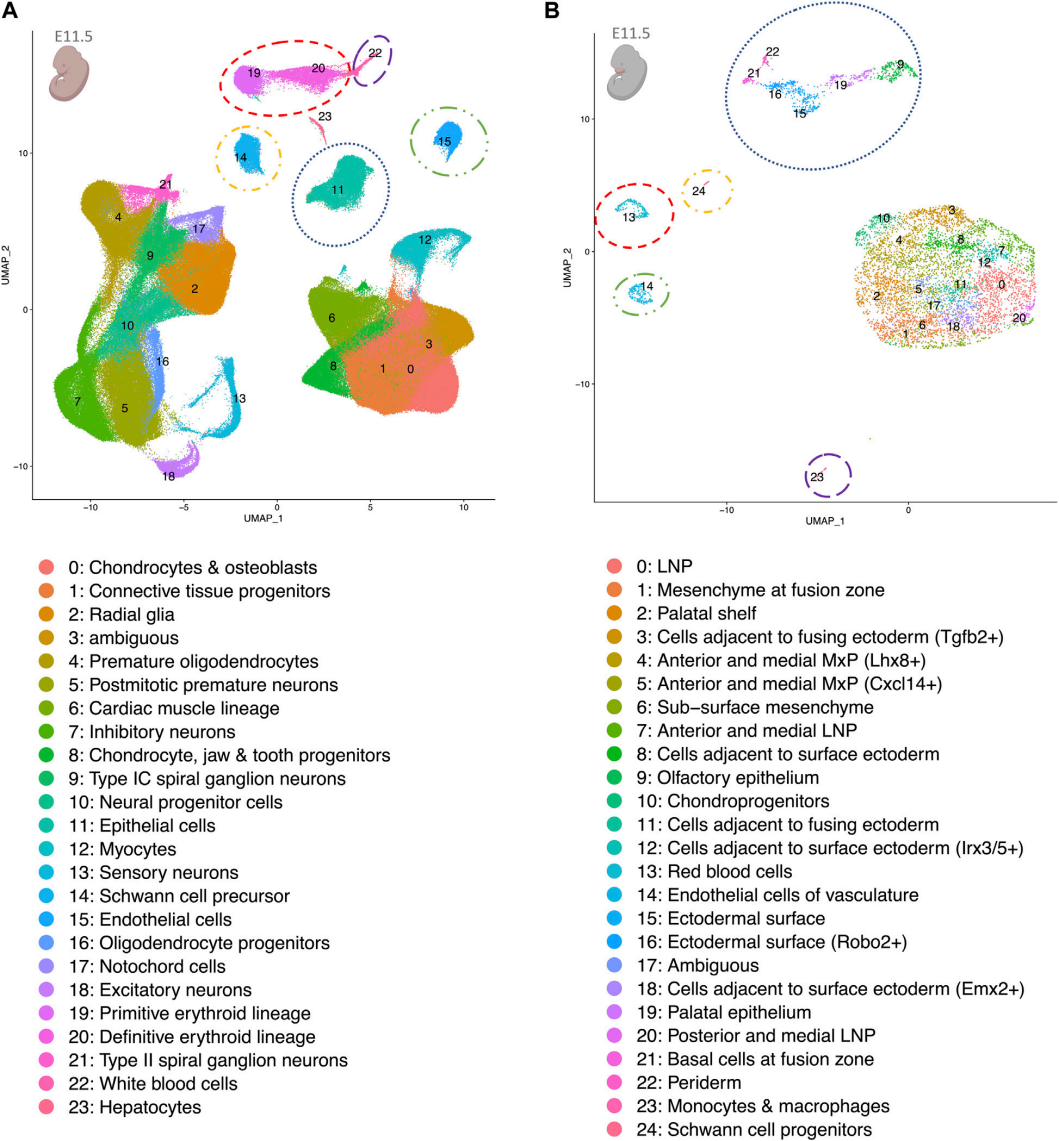
UMAP plots of re-analyzed scRNA-seq whole embryo data at E11.5 **(A)** and facial data at E11.5 **(B)**. Despite differing read depths in the two data sets, shared cell clusters corresponding to matched cell types are observed. These are encircled in the same color in both panels. The pink colors of the embryo graphics correspond to the tissues that are included in the data set. Lateral nasal process (LNP), maxillary prominence (MxP).

Figure 2 shows the results generated by the UMAP algorithm for both the facial data (panel B) and the MOCA data (panel A, E11.5, all other time points in Supplementary Figures S1A–D). The 25 cell types observed in the facial data were grouped into two main cell type clusters: (i) epithelial cells comprising periderm, basal cells at fusion zone, ectodermal surface, ectodermal surface (*Robo2*+), olfactory epithelium, and palatal epithelium; and (ii) more diverse cell types, which share a mesenchymal state, as based on the analysis of mesenchymal cell markers (Supplementary Figure S1E). Smaller cell clusters included endothelial cells and Schwann cells (Figure 2B). In the MOCA data for E11.5, a total of 24 cell types were identified, including a distinct epithelial cluster. To determine whether this at least partially represents the epithelial clusters in the facial data, the epithelial cells were sub-clustered. Three of these sub-clusters express marker genes for periderm (sub-cluster 6), basal cells at the fusion zone (sub-cluster 7) and ectodermal surface (sub-clusters 8 & 9) (Supplementary Figure S1G; marker genes of the sub-clusters in Supplementary Table S3). Additional cell clusters in MOCA comprised specific cell types, such as hepatocytes, which are not represented in the facial data, as well as overlapping cell types where expected, e.g., endothelial cells, Schwann cells, and red and white blood cell types (Figure 2A, B colored circles).

### 3.2 A subset of CL/P candidate genes show convergent expression patterns

**FIGURE 3:**
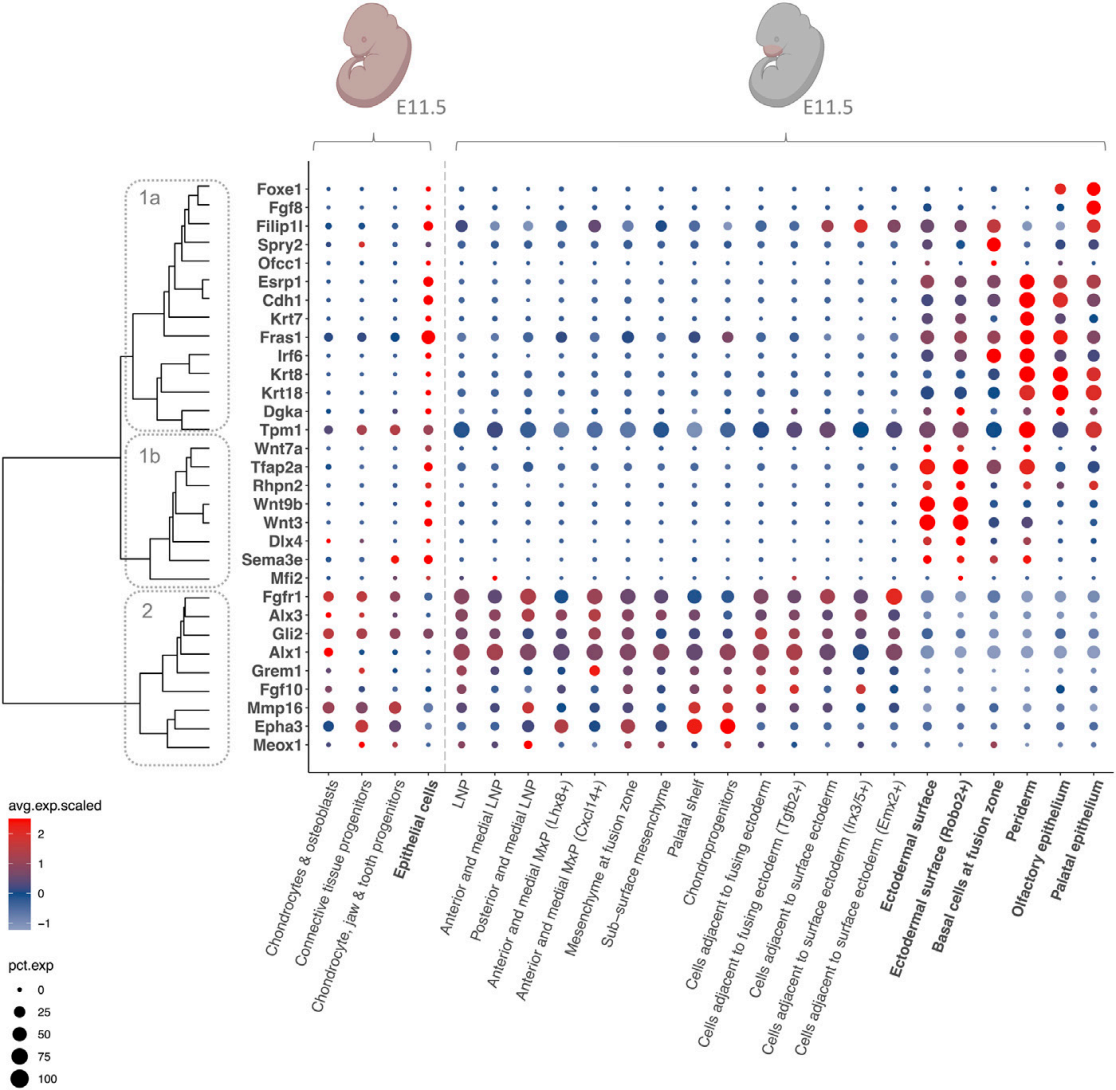
Expression patterns of CL/P candidate genes at E11.5. Dotplot of gene expression for selected CL/P candidate genes at E11.5 in selected cell types in the MOCA and the facial data. The color of the dots corresponds to the average scaled expression level. The size of the dots corresponds to the percentage of cells that express the gene in the respective cell type. Epithelial cell types are indicated in bold, and mesenchymal-like cell types are indicated in non-bold. Dendrogram cluster 1a = genes expressed predominantly in periderm, basal cells at fusion zone, olfactory epithelium, and palatal epithelium; cluster 1b = genes predominantly expressed in ectodermal surface; cluster 2 = genes expressed predominantly in mesenchymal-like cell types. The pink colors of the embryo graphics correspond to the tissues that are included in the data set. Lateral nasal process (LNP), maxillary prominence (MxP).

Investigation of the expression patterns of CL/P candidate genes in the scRNA-seq data sets showed that while the facial data set allowed an in-depth investigation of craniofacial structures at E11.5, the MOCA data set enabled a time course analysis over the time span of craniofacial development. Of the 126 CL/P candidate genes, all were expressed in the MOCA data sets from E9.5 - E13.5, although they varied in terms of overall expression levels and the cell types in which they were expressed. In the MOCA data, many CL/P candidate genes showed ubiquitous expression at E9.5, which became more specific at E10.5. Among the 126 CL/P candidate genes, 31 were specifically expressed in cell types of relevance to craniofacial development (i.e., epithelial cells, chondrocytes and osteoblasts, connective tissue progenitors, chondrocyte and jaw and tooth progenitors). Here, “specific expression” refers to either: (i) expression in at least one of these cell types; or (ii) expression in additional cell types, but with the highest expression levels being observed in at least one of the cell types of relevance to craniofacial development. Comparison of the expression patterns of these 31 genes in the MOCA and the facial data (Figure 3) revealed that they clustered into two main groups: While 22 genes were specifically expressed in epithelial cell types (Figure 3 dendrogram cluster 1), nine genes were expressed in mesenchymal-like cell types (Figure 3 dendrogram cluster 2). Interestingly, the analyses showed that the first group (i.e., genes expressed predominantly in epithelial cell types) can be further subdivided into genes that have their highest expression levels in the ectodermal surface (Figure 3 dendrogram cluster 1b), and genes that have their highest expression levels in periderm, basal cells at fusion zone, olfactory epithelium, and palatal epithelium (Figure 3 dendrogram cluster 1a). The expression patterns of the remaining 95 CL/P candidate genes at E11.5 are shown in Supplementary Figure S4.

### 3.3 CL/P may involve distinct subgroups of epithelial cells

Using our data set, we first focused on the well-established CL/P risk gene *IRF6*. In the present study, *Irf6* was predominantly expressed in epithelial cells in both the MOCA and the facial data sets, with particularly strong expression being observed in the periderm and basal cells at fusion zone in the facial data set. In MOCA, this expression was maintained throughout the developmental time period of the data set (Supplementary Figure S3). In the facial epithelial cells, considerable intra-cluster heterogeneity was observed. Cells expressing *Irf6* (denoted as *Irf6*+ cells) were observed in 58% of cells from the palatal epithelium (*n* = 71 out of 170 cells), 40% of cells from olfactory epithelium (105/258), 44% of cells from the ectodermal surface (85/192), 44% of cells from the ectodermal surface (*Robo2*+) (86/192), 70% of cells from the basal cells at fusion zone (49/70), and 77% of the periderm cells (41/53). The six epithelial cell clusters from the facial data set were each divided into subsets according to their expression of *Irf6*, and marker genes of the *Irf6*+ cells were identified (Supplementary Table S3). A set of genes that overlapped between the marker genes of the *Irf6*+ epithelial subsets and CL/P candidate genes was identified (Table 1), which included CL/P genes that were associated with: (i) syndromic forms (e.g., *Tfap2a* (Branchio-oculo-facial syndrome, Milunsky et al., 2008), *Ctnnd1* (Blepharocheilodontic syndrome 2, Ghoumid et al., 2017) and *Fras1* (Fraser syndrome 1, Fraser, 1962); and (ii) candidate genes from GWAS loci (e.g., *Tpm1* (Ludwig et al., 2012) and *Arid3b* (Leslie et al., 2017) (Table 1, Supplementary Table S4). Interestingly, the gene Grainyhead-like 3 (*Grhl3*) was also observed among the marker genes of cells from the periderm and olfactory epithelium. As with *Irf6*, mutations in *Grhl3* cause Van der Woude syndrome. Here, however, most individuals present with a cleft palate only rather than CL/P (Mangold et al., 2016).

**TABLE 1:** CL/P candidate genes with specific expression in *Irf6*+ facial epithelial cells.^1^ adjusted *p*-value (based on Bonferroni correction using all genes in the data set);^2^ average log_2_ fold change in the average expression between the two tested groups (second test group: all other cell types; positive values indicate that the gene is more highly expressed in the respective cell type compared to all other cell types). NsCLO (non-syndromic cleft lip only).

Cell type	Gene	P-val. adj.^1^	Log2FC^2^	Cleft association in humans
**Periderm**	*Tpm1*	1.9E-09	1.08	nsCL/P GWAS (Ludwig et al., 2012)
	*Pik3r1*	6.1E-10	0.75	nsCL/P GWAS (Leslie et al., 2017)
	*Tfap2a*	4.9E-44	1.03	Branchio-oculo-facial syndrome (Milunsky et al., 2008), nsCL/P GWAS (Ludwig et al., 2012; Leslie et al., 2017)
	*Wnt3*	1.0E-10	0.25	Tetra-amelia syndrome 1 (Niemann et al., 2004)
	*Ctnnd1*	0.0002	0.42	Blepharocheilodontic syndrome 2 (Ghoumid et al., 2017)
	*Fras1*	8.0E-10	0.69	Fraser syndrome (Fraser, 1962)
**Basal cells at fusion zone**	*Spry2*	4.0E-37	1.29	nsCL/P GWAS (Ludwig et al., 2012)
**Ectodermal surface**	*Arid3b*	7.7E-13	0.3	nsCL/P GWAS (Leslie et al., 2017)
	*Zfp36l2*	0.008	0.26	nsCL/P (Lin-Shiao et al., 2019), nsCLO (Li et al., 2022)
**Ectodermal surface (*Robo2*+)**	*Tpm1*	0.04	0.3	nsCL/P GWAS (Ludwig et al., 2012)
**Palatal epithelium**	*Cyb561*	1.6E-36	0.3	nsCL/P GWAS (Leslie et al., 2016)
	*Ptch1*	0.0001	0.33	nsCL/P GWAS (Yu et al., 2017), CPO GWAS (Butali et al., 2019), Basal cell nervous syndrome (Evans et al., 1993; Kimonis et al., 1997; Kimonis et al., 2013)
	*Tfap2a*	1.9E-09	0.27	Branchio-oculo-facial syndrome (Milunsky et al., 2008), nsCL/P GWAS (Ludwig et al., 2012; Leslie et al., 2017)
	*Fras1*	2.0E-08	0.33	Fraser syndrome (Fraser, 1962)
	*Ripk4*	5.3E-32	0.3	Popliteal pterygium syndrome, Bartsocas-Papas type 1 (Bartsocas and Papas, 1972)
**Olfactory epithelium**	*Arid3b*	2.5E-11	0.25	nsCL/P GWAS (Leslie et al., 2017)
	*Cyb561*	5.0E-55	0.29	nsCL/P GWAS (Leslie et al., 2016)
	*Xpa*	0.0003	0.26	nsCL/P GWAS (Welzenbach et al., 2021)

**FIGURE 4:**
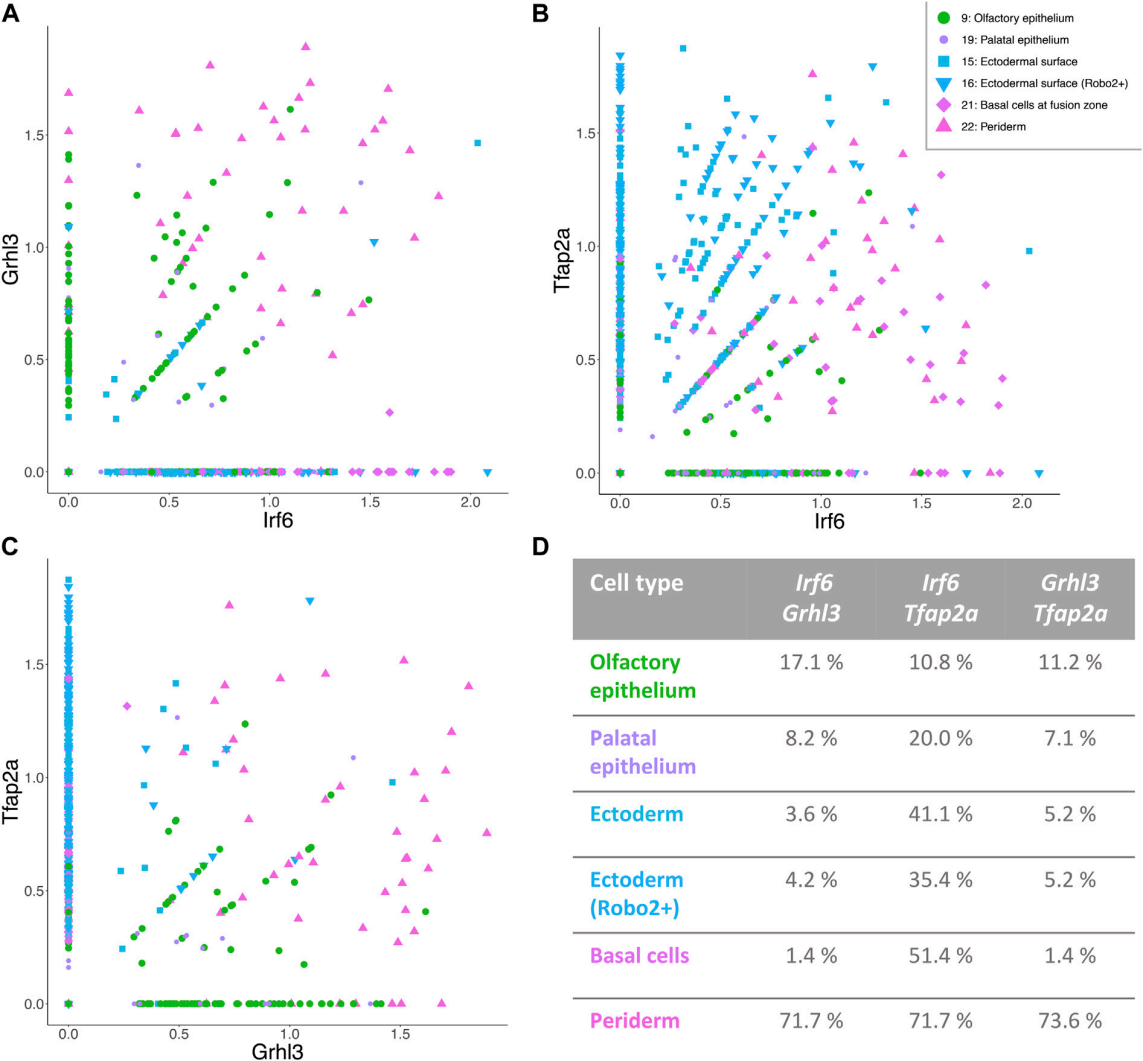
Distinct populations of epithelial cells with a possible involvement in CL/P. **(A–C)** *Irf6-Grhl3-Tfap2a* show partial co-expression in epithelial cell types of E11.5 facial data. The axes of the graphs represent the expression level. Legend for all three figures is positioned in panel **(B)**. **(D)** Table showing the percentage of cells with co-expression of the respective gene pair in all six epithelial cell clusters.

To elucidate the connection of *Irf6*, *Grhl3,* and *Tfap2a* in the six epithelial cell types at the transcriptomic level, the co-expression of these genes was analyzed (Figures 4A–D). Each of the *Irf6-Grhl3-Tfap2a* gene pairs showed partial co-expression, since an overlap in expression was observed in a subgroup of cells (indicated by percentage in Figure 4D). The co-expression network comprising all three genes was most abundant in the periderm, while it was reduced to only *Irf6* and *Tfap2a* in basal cells at fusion zone, the ectodermal surface clusters, and the palatal epithelium as well.

### 3.4 SyCL/P genes are expressed in more tissues compared to nsCL/P genes

**FIGURE 5:**
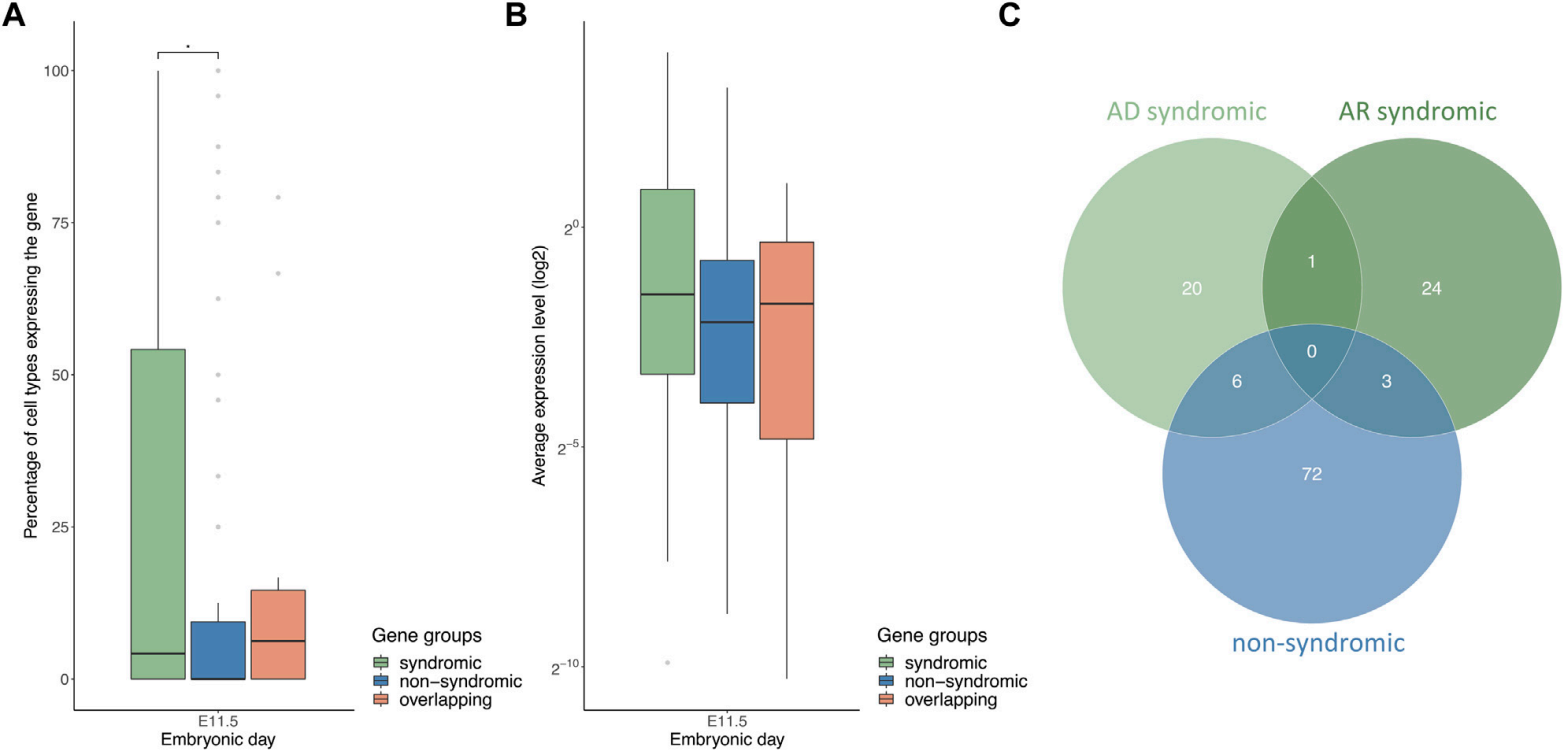
AD syCL/P genes are expressed in more cell types and have higher average expression levels compared with nsCL/P genes. **(A)** Boxplot of the percentages of cell types expressing the gene groups of syCL/P, nsCL/P, and overlapping genes at E11.5 **(B)** Boxplot of average log2 expression levels of the gene groups of syCL/P, nsCL/P, and overlapping genes at E11.5. **(C)** Venn diagram of non-syndromic, AR syndromic, and AD syndromic CL/P gene lists. (*p < 0.05). Data on the remaining time points are provided in Supplementary Figure S2.

To compare differences in the number of cell types between the gene lists for syCL/P and nsCLP, the analysis was restricted to the MOCA data set only, since syCL/P can affect tissues and organs outside of the craniofacial region and the MOCA data set contains more non-facial tissues. Across stages E10.5 to E11.5, the syCL/P genes were expressed in significantly more cell types than was the case for the nsCL/P genes (Figure 5A, E11.5). Comparison of the average gene expression levels of these gene sets showed that the syCL/P genes did not have significantly higher gene expression levels than the nsCL/P genes (Figure 5B E11.5). However, division of the syCL/P gene set into AD and AR genes revealed that the observed differences in the percentage of expressing cell types between syCL/P and nsCL/P were mainly driven by the AD syCL/P genes. In comparison to the nsCL/P and AR syCL/P genes, the AD syCL/P genes: (i) were expressed in more cell types (Supplementary Figure S2A); and (ii) showed higher average expression levels (Supplementary Figure S2B).

## 4 Discussion

The present study leveraged two scRNA-seq data sets to generate insights into craniofacial development and diseases, specifically CL/P. Our reasons for selecting these data sets were threefold. First, the process of craniofacial development is largely conserved between mice and humans (Suzuki et al., 2016), which suggests that murine scRNA-seq data can be useful in terms of studying craniofacial development in the absence of human data. Second, the respective scRNA-seq samples were obtained at the time period of murine primary and secondary palate development (Miyake et al., 1996), thus increasing their suitability for studying CL/P candidate genes. Finally, research has shown that a large proportion of human embryonic scRNA-seq data from later developmental time points can be integrated with the MOCA data (Cao et al., 2020), providing further evidence for the transferability of developmental expression patterns. Although the MOCA scRNA-seq data are easily accessible via a comprehensive web browser, a systematic analysis in this setting is challenging. Of the 38 major cell clusters originally reported in MOCA, the present re-analysis identified a total of 31. This was probably attributable to differences in processing, since in the present study, the data were first split in accordance with embryonic day (in order to reduce the size of the data set to a computable level), followed by the performance of clustering. Nevertheless, as in the original MOCA publication, less diffuse clustering of some cell types was observed over the 5 day time-period, and a joint clustering of mesenchymal-like cell types was identified, such as chondrocyte progenitors, connective tissue progenitors, chondrocytes and osteoblasts, and jaw and tooth progenitors (commencing at E10.5). With regards to the facial dataset, the present analysis identified 25 clusters as opposed to 24 main clusters reported in the original publication. While we consider the numbers of clusters similar, we observed differences in cluster annotations. On one side, our re-analysis yielded several distinct cluster annotations for four clusters that were annotated as one cluster each in Li et al. This increased the number of clusters comprising those cells. On the other hand, we also failed to identify four of the 24 original clusters, including nasolacrimal groove and dental epithelium (see Supplementary Table S1). Investigating this further, we identified marker genes for these two clusters to be predominantly expressed in some of the cells of our ectodermal surface clusters and palatal epithelium, respectively (Supplementary Figure S5). Yet, these clusters did not split further into distinct clusters when using higher resolution clustering (*data not shown*). This divergence may be attributable to the fact that the present analysis involved a stricter filtering strategy, no cell cycle regression, and high-resolution clustering of all cells together without sub-clustering (as opposed to the original study that divided the data into ectoderm and mesenchyme first, and performed sub-clustering at different resolutions individually). Together, this contributed to a lower absolute number of overall cells (7,249 compared to 7,893 from the original) and different clusters in our re-analysis.

Comparison of E11.5 transcriptome profiles between the MOCA and the facial data revealed substantial similarities at both the cell type and gene levels. For instance, red and white blood cells, endothelial cells, and Schwann cells represent distinct cell clusters that mapped at certain distances to the other clusters within the UMAP space. At the gene level, *Irf6*, *Tfap2a*, *Fras1*, *Cdh1,* and *Esrp1* exhibited similar expression patterns in epithelial cell types of both data sets. Additionally, *Tfap2a* showed expression in Schwann cell progenitors in both data sets. Together, these data suggest that the facial data set is a tissue-restricted, but high-resolution representation of the MOCA data at E11.5, and that collectively, the two datasets represent a valuable resource for genomics research into craniofacial development. However, caution is generally required when interpreting expression profiles from several scRNA-seq data sets, since scRNA-seq itself but also the combination of different sources have some limitations. These include differences in cell capture efficiency and transcript coverage, which may result in transcripts not being detected in all cells equally, and different enrichment strategies used in both studies. In addition, scRNA-seq data of tissues undergoing continuous processes during development, such as epithelial-to-mesenchymal transitions, only provide a snapshot of a possibly transient period of gene expression. Finally, varying sequencing depth adds to higher noise levels in scRNA-seq data compared to bulk RNA-seq data (Kolodziejczyk et al., 2015).

The expression patterns observed in the aggregated scRNA-seq data sets replicate previously reported and experimentally verified expression patterns. For instance, a previous study showed that *Irf6* is expressed in neural ectoderm and neural crest cells as early as E9.5 in murine embryonic development (Kousa et al., 2019). According to previous wet-lab data, *Irf6* is expressed in the ectoderm of the first and second pharyngeal arches, and in the palatal, lingual, maxillary, and mandibular epithelia, during the period E10.5–E13.5 (Kondo et al., 2002; Knight et al., 2006; Richardson et al., 2009; Goudy et al., 2013; Kousa and Schutte, 2016). In accordance, our analyses revealed the presence of *Irf6* expression in Schwann cell precursors, the palatal and olfactory epithelia, the ectodermal surface, the basal cells at the fusion zone, and the periderm in both, MOCA and facial data respectively. Of these, the highest expression was observed in the periderm and the basal cells at the fusion zone. Interestingly, only ∼3% of the MOCA E11.5 epithelial cells expressed *Irf6*, as opposed to 40%–70% of those in the facial data set. This suggests that *Irf6*-expressing MOCA E11.5 epithelial cells might be derived from facial structures, while the epithelial cell cluster contains a substantial proportion of non-facial cells. Comparably, *Tfap2a* showed expression in the MOCA epithelial cells, as well as high expression levels in the facial ectodermal surface clusters and periderm. In addition, *Tfap2a* showed expression in Schwann cell precursor cells in both the MOCA and the facial data sets. Again, this expression pattern recapitulates existing data, since previous reports have demonstrated that in mice, *Tfap2a* is expressed in the ectoderm, cranial neural crest cells, the facial mesenchyme, nasal and oral epithelia, and the central and peripheral nervous system between E9–E13.5 (Mitchell et al., 1991; Chazaud et al., 1996; Moser et al., 1997). Previous studies have shown that *Esrp1* is expressed in the head region and epithelial cells, especially in cells of the ectodermal surface as early as E9.5 in mice (Warzecha et al., 2009; Revil and Jerome-Majewska, 2013; Bebee et al., 2015; Lee et al., 2020). Similarly, our data showed a broad expression of *Esrp1* in epithelial cells of both data sets with the highest expression in the periderm in the facial data set. Furthermore, the transcription factor *Foxe1* was found to be expressed in epithelial cells of embryonic mice starting at E9.5, both in our data sets and in previous studies (Zannini et al., 1997; Dathan et al., 2002; Moreno et al., 2009). In addition, studies have shown the keratin genes *Krt8* and *Krt18* to be expressed in single-layered epithelia in embryos (Jackson et al., 1980; Owens and Birgitte Lane, 2003; Moll et al., 2008). This is also confirmed by our data, as *Krt8* and *Krt18* showed expression in epithelial cells in both data sets. However, as expected, the strongest expression in the facial data set was found in the periderm and the palatal and olfactory epithelia. In contrast to the previously described genes, *Fgfr1* has been shown to be primarily expressed in mesenchymal cell types (Bachler and Neubüser, 2001), whis is also evident in our data, as *Fgfr1* was predominantly expressed in mesenchymal cell types. These similarities indicate that: (i) the data sets are reliable resources in the context of craniofacial development; and (ii) that expression patterns of genes that have not yet been experimentally validated may be characterized using scRNA-seq data.

In a first attempt to use these data in the context of CL/P, the present analyses identified two groups of CL/P candidate genes based on their expression in relevant facial cell types. Using predefined lists of CL/P candidate genes, the analyses identified distinct sets of genes that are predominantly expressed in either epithelial cells, or mesenchymal-like cells. Unsurprisingly, the first group included *Irf6*, *Tfap2a,* and *Esrp1*, which show similar expression in the six epithelial cell types of the facial data set, and which have been implicated in a regulatory network (Kousa et al., 2019; Carroll et al., 2020). A specific examination of the expression of the *Irf6*-*Grhl3*-*Tfap2a* genetic pathway revealed partial co-expression of *Irf6*, *Grhl3,* and *Tfap2a* within epithelial cells. This opens up the possibility that other CL/P candidate genes, which are among the marker genes of the *Irf6*+ epithelial cell types, or genes with an as yet unknown role in CL/P etiology, might also contribute to the *Irf6* regulatory network. We plan to follow up on this question in a future study, using more systematic co-expression network approaches (Dam et al., 2018). While the expression of *Irf6* in the periderm has already been established (Richardson et al., 2009; Richardson et al., 2014; Kousa et al., 2017), the scRNA-seq data suggest the presence of a specific sub-cell type in which *Irf6* and other CL/P candidate genes show co-expression, and that may contribute to the etiology of CL/P. Furthermore, the expression of CL/P candidate genes in adjacent facial cell types highlights CL/P candidate genes that might contribute to molecular communication between the different epithelial cell types, e.g., *Tpm1, Fras1, Krt7, Wnt7a, Rhpn2,* and *Sema3e* in the ectodermal surface clusters and periderm; and *Filip1l* in the ectodermal surface and cells adjacent to the ectodermal surface. These questions need to be addressed in the future using more sophisticated computational and experimental approaches, such as spatial transcriptomic analyses (Carangelo et al., 2022).

In a second application example, the MOCA data set was used to investigate potential differences in expressing cell types between syCL/P and nsCL/P candidate genes. In accordance with our hypothesis, syCL/P candidate genes were expressed in a larger number of cell types during the examined time period compared to candidate genes for nsCL/P. Similar patterns were observed in the analysis of the gene lists for CHD. The AD syndromic CHD genes were expressed in significantly more cell types than the AR syndromic and the non-syndromic CHD genes (Supplementary Figure S2C). The average expression levels of the AD syndromic CHD genes were significantly higher than those of the non-syndromic CHD genes (Supplementary Figure S2D). While the precise reason for this effect requires further investigation, our analysis indicates the value of scRNAseq data in terms of the investigation of the different genetic architectures of CL/P subtypes.

In summary, the present study involved a re-analysis of previously published scRNA-seq data. We demonstrate the value of these data using several application examples. Our processed data sets are provided in Seurat object format as an easily accessible addition to the original data (see “Data availability statement”). This resource will facilitate functional approaches to the genomics of craniofacial development and disease.

## Data Availability

The original contributions presented in the study are included in the article/Supplementary Material, the processed data sets can be downloaded from https://beta.fastgenomics.org/p/Siewert_2023. Further inquiries can be directed to the corresponding author.
