# Supplementary material for: Analysis of candidate genes for cleft lip ± cleft palate using murine single-cell expression data
Source: Front Cell Dev Biol. 2023 Apr 24;11:1091666. doi: 10.3389/fcell.2023.1091666 (PMC10165499; doi:10.3389/fcell.2023.1091666)
Supplement: Supplementary file 2 [file DataSheet1.docx]

Supplementary Material

# Supplementary Figures and Tables

**Supplementary Figure 1.** UMAP plots of MOCA data at E9.5 (A), E10.5 (B), E12.5 (C), and E13.5 (D). (E) Dotplot of expression of mesenchymal markers (published in Li et al. 2019) in selected cell types at E11.5 in the facial data. (F) Dotplot of expression of mesenchymal markers (same as in E) in selected cell types at E11.5 in the MOCA data. *Creb5* was not present in the data set. (G) Dotplot of epithelial cell type marker (published in Li et al. 2019) expression in subclusters of epithelial cells from the MOCA E11.5 data set. Subcluster 6 expresses marker genes of the periderm, subcluster 7 expresses marker genes of the basal cells at fusion zone, and subcluster 8 and 9 express marker genes of the ectodermal surface.

**Supplementary Figure 2.** (A) Comparison of cell types expressing syCL/P, nsCL/P, and overlapping genes in the MOCA E9.5 - E13.5 expression data. (B) Comparison of average log2 expression levels of syCL/P, nsCL/P, and overlapping genes in the MOCA E9.5 - E13.5 expression data. (C) Boxplot of the percentages of cell types expressing the gene groups of AD syCHD, AR syCHD, and nsCHD at E9.5 - E13.5. (D) Boxplot of average log2 expression levels of the gene groups ofAD syCHD, AR syCHD, and nsCHD at E9.5 - E13.5. (* P<0.05, ** P ≤ 0.01, *** P ≤ 0.001, **** P ≤ 0.0001).

**Supplementary Figure 3.** Dotplots of all CL/P candidate genes from Figure 3 from MOCA E9.5 (A), E10.5 (B), E11.5 (C) , E12.5 (D), E13.5 (E), and facial data from E11.5 (F).

**Supplementary Figure 4.** Dotplot of all remaining 95 CL/P candidate genes in MOCA E11.5 data.

**Supplementary Figure 5.** UMAP plots of facial data set with cells colored in expression level of the respective gene. (A) Marker genes for nasolacrimal groove. (B) Marker genes for dental epithelium (Shh, Lmo2, Fgf8) and anterior oral cavity (Shh). Marker gene information from Li et al. 2019.
